# Anticancer properties and metabolomic profiling of *Shorea roxburghii* extracts toward gastrointestinal cancer cell lines

**DOI:** 10.1186/s12906-024-04479-1

**Published:** 2024-04-30

**Authors:** Sutthiwan Janthamala, Bundit Promraksa, Malinee Thanee, Kunyarat Duenngai, Apinya Jusakul, Sarinya Kongpetch, Ratthaphol Kraiklang, Kidsada Thanee, Porntip Pinlaor, Nisana Namwat, Hideyuki Saya, Anchalee Techasen

**Affiliations:** 1 Biomedical Sciences Program, Graduate School, Khon Kaen University, Khon Kaen, Thailand; 2 Regional Medical Sciences Center 2 Phitsanulok, Department of Medical Sciences, Ministry of Public Health, Phitsanulok, Thailand; 3 Department of Pathology, Faculty of Medicine, Khon Kaen University, Khon Kaen, Thailand; 4 Department of Thai Traditional Medicine, Faculty of Science and Technology, Phetchabun Rajabhat University, Phetchabun, Thailand; 5 Centre for Research and Development of Medical Diagnostic Laboratories (CMDL), Faculty of Associated Medical Sciences, Khon Kaen University, Khon Kaen, Thailand; 6 Department of Pharmacology, Faculty of Medicine, Khon Kaen University, Khon Kaen, Thailand; 7 Nutrition for Health Program, Faculty of Public Health, Khon Kaen University, Khon Kaen, Thailand; 8 Faculty of Public Health, Ubon Ratchathani Rajabhat University, Ubon Ratchathani, Thailand; 9 Systems Biosciences and Computational Medicine, Faculty of Medicine, Khon Kaen University, Khon Kaen, Thailand; 10 Cancer Center, Fujita Health University, Toyoake, Aichi Japan

**Keywords:** Gastrointestinal cancer, Medicinal plant, Antioxidant, Anticancer, Metabolic profiling-based screening

## Abstract

**Background:**

Gastrointestinal cancer (GIC) ranks as the highest cause of cancer-related deaths globally. GIC patients are often diagnosed at advanced stages, limiting effective treatment options. Chemotherapy, the common GIC recommendation, has significant disadvantages such as toxicity and adverse effects. Natural products contain substances with diverse pharmacological characteristics that promise for use in cancer therapeutics. In this study, the flower of renowned Asian medicinal plant, *Shorea roxburghii* was collected and extracted to investigate its phytochemical contents, antioxidant, and anticancer properties on GIC cells.

**Methods:**

The phytochemical contents of *Shorea roxburghii* extract were assessed using suitable methods. Phenolic content was determined through the Folin-Ciocalteu method, while flavonoids were quantified using the aluminum chloride (AlCl_3_) method. Antioxidant activity was evaluated using the FRAP and DPPH assays. Cytotoxicity was assessed in GIC cell lines via the MTT assay. Additionally, intracellular ROS levels and apoptosis were examined through flow cytometry techniques. The correlation between GIC cell viability and phytochemicals, ^1^H-NMR analysis was conducted.

**Results:**

Among the four different solvent extracts, ethyl acetate extract had the highest phenolic and flavonoid contents. Water extract exhibited the strongest reducing power and DPPH scavenging activity following by ethyl acetate. Interestingly, ethyl acetate extract demonstrated the highest inhibitory activity against three GIC cell lines (KKU-213B, HepG2, AGS) with IC_50_ values of 91.60 µg/ml, 39.38 µg/ml, and 35.59 µg/ml, while showing less toxicity to normal fibroblast cells. Ethyl acetate extract induced reactive oxygen species and apoptosis in GIC cell lines by downregulating anti-apoptotic protein Bcl-2. Metabolic profiling-based screening revealed a positive association between reduced GIC cell viability and phytochemicals like cinnamic acid and its derivatives, ferulic acid and coumaric acid.

**Conclusions:**

This study highlights the potential of natural compounds in *Shorea roxburghii* in the development of more effective and safer anticancer agents as options for GIC as well as shedding light on new avenues for cancer treatment.

**Supplementary Information:**

The online version contains supplementary material available at 10.1186/s12906-024-04479-1.

## Background

Gastrointestinal cancer (GIC) is defined as the cancer of organs of the digestive system [1]. According to Cancer Statistics 2021, GIC is ranked the highest in cancer-related deaths (28%) and second in all new cancer cases (18%) [2]. Although genetic predisposition and lifestyle habits have been considered to be the most important risk factors for GIC development, chronic inflammation is another factor that causes GIC [1, 3]. Pathogen infections such as *Opisthorchis viverrini*, *Helicobacter pylori*, as well as viral hepatitis B and C leading to the increasing of oxidative stress which can damage biomolecules, triggering mutations, eventually leading to cancer development [4–8]. Surgery, radiotherapy, and chemotherapy, remain to be the main current conventional therapies for cancer treatment [9, 10]. However, chemotherapy which is the major conventional therapy for GIC patients in the advanced stages is costly, has many adverse effects, and increases drug resistance [11–14]. The discovery of new anticancer agents which are simple and have fewer side effects is the challenge in cancer treatment.

Medicinal plants and plant-derived products have been shown to be an important source of anti-cancer agents because they are simple, safer, eco-friendly, low-cost, and less toxic than traditional treatment approaches [15–18]. Medicinal plants contain various phytochemical compounds, which current technology has identified many effective anticancer substances, for example, polysaccharides, triterpenes, flavonoids, proteins. Several chemotherapeutic agents have been derived from natural extracts, herbal medicine and purified bioactive compounds. These agents have received approval from the US Food and Drug Administration (FDA) [19–22]. Paclitaxel (Taxol^®^) is a broad-spectrum anticancer compound which isolated from the bark of the yew tree *Taxus brevifolia* Nutt. It has been licensed by the FDA for the treatment of metastatic ovarian cancer since 1992. Clinical studies have shown promising outcomes on paclitaxel treatment in other cancer types, including head and neck, lung, and breast cancers [23–30]. Camptothecin (CPT), a quinoline alkaloid was discovered in 1966 from the stem of *Camptotheca acuminate*, a Chinese ornamental tree. It is the main molecule of a significant family of medicines that target the nuclear enzyme topoisomerase I. Camptothecin and its clinical derivatives are currently among the most effective anti-cancer treatments for advanced-stage gastrointestinal, ovarian, and recurrent small-cell lung cancers [31, 32]. Salvestrols are resveratrol derivatives which a category of naturally occurring anticancer plant metabolites identified in 1998. It behaves as a prodrug when activated by the cytochrome P450 enzyme CYP1B1. Specifically, CYP1B1 metabolizes salvestrol to produce a compound that causes apoptosis within the cancer cell [33, 34]. Artesunate, derived from *Artemisia annua*, induces ROS-independent apoptosis in HepG2 cells. This finding suggests that artesunate has potential as cancer treatment [35]. Clinical trials confirm its safety, recommending doses up to 200 mg/d for further trials [36]. Preclinical investigations have shown that curcumin, derived from turmeric (*Curcuma longa*), has anticancer properties. When used in combination with gemcitabine, it is considered safe for pancreatic cancer patients [37]. Resveratrol, mainly in grapes, regulates key cancer pathways. The phase I trials with SRT501 in colorectal cancer demonstrate both tolerance and increase in apoptotic markers [38–41]. In addition, Mustafa et al. demonstrated that water/ethanolic extract of *Berberis lycium* Royle (BLE) significant inhibited HepG2 cancer cell line growth and triggered apoptotic cell death through a reduction in Bcl-2 [42]. Berberine, an isoquinoline alkaloid extracted from plants has the growth inhibition effect, attenuated the invasion and migration on AGS and SGC7901 cancer cell lines, and suppressed the tumor growth in mouse models [43, 44]. The growth of colorectal adenocarcinoma HT-29 cells was inhibited by treatment with an extract from *Chrysobalanus icaco*, which is rich in anthocyanins. This treatment led to an increase in intracellular ROS production [45].

*Shorea roxburghii* is a large perennial plant which is found in all regions in Thailand and the neighboring countries [46]. It has stem bark and fragrant flowers that have been employed as a preservative for traditional beverages, and used for treating dysentery, diarrhea [47]. The extracts from bark of *Shorea roxburghii* have a large number of polyphenols which are phytochemical substances such as hopeaphenol, resveratrol, and hemsleyanol D. It has been shown that these contain significant antioxidant, cytotoxic, and anticancer properties [46, 48–50]. Recent reports indicate that several oligostilbenoids have greater anti-proliferative effects compared to the comparable monomer, trans-resveratrol, against human malignant melanoma cells [48]. The polyphenols found in the bark and wood of *Shorea roxburghii* shown hepatoprotective properties against liver injury induced by D-galactosamine /lipopolysaccharide in mice [50]. However, few studies have examined the anti-cancer properties of this medicinal flower.

The traditional approach to natural product-based therapeutic development begins with a biological screening of crude extracts to discover a bioactive component. A metabolic profiling-based technique provides accurate information into the metabolite composition found in natural product extracts. This allows the identification of novel bioactive compounds derived from natural sources. Current metabolomics strategies are mainly reliant on two major platforms: Mass spectrometry (MS), and nuclear magnetic resonance (NMR) spectroscopy [51]. NMR spectroscopy is a powerful analytical tool for metabolic profiling which is simple, fast, low cost per sample, and more advantageous [52].

Therefore, the aim of this study was to examine the phytochemical contents, antioxidant properties, and assess the anticancer potential of *Shorea roxburghii* extracts on gastrointestinal cancer cell lines. Additionally, ^1^H-NMR was applied to identify potentially active anticancer compounds derived from *Shorea roxburghii* that could be used for gastrointestinal cancer treatment.

## Methods

### Herbal plant collection and identification

The *Shorea roxburghii* obtained with permission was harvested from our team's field, Dr. Malinee Thanee in Ubon Ratchathani province, Thailand in June 2021. The plant was authenticated by Dr. Sukanya Dej-adisai, Department of Pharmacognosy and Pharmaceutical Botany, Faculty of Pharmaceutical Sciences, Prince of Songkla University, Songkhla, Hat Yai, Thailand. A voucher specimen (SKP 064 19 18 01) has been deposited in the herbarium of our institute, Faculty of Associated Medical Sciences, Khon Kaen University, Khon Kaen, Thailand for future reference.

### Sample preparation and crude extraction

The flower of *Shorea roxburghii* was dried at room temperature. The dried plant was ground into a fine powder (450 g). The powder was macerated for three days in each series solvent. Hexane (HEX) was used as the first solvent, with a total volume of 4.5 L, at room temperature. After three days, filter the liquid through two layers of gauze and no. 1 Whatman paper. A rotary evaporator R-100 (Buchi, Switzerland) was used to evaporate the liquid extract under vacuum. The residue was air dried and further extracted with ethyl acetate (EtAc) and following by ethanol (EtOH), and distilled water (Water) similar to the procedure carried out for the HEX extract. The crude extracts of *Shorea roxburghii* were stored at -20 °C with light protection for further analyses.

### Total phenolic contents

The total phenolic content of the extracts was determined by the Folin-Ciocalteu method. The experiment was adapted from a previous study [53]. Briefly, 20 µl of extract (1 mg/ml) solution was mixed with 100 µl of 10% (w/v) Folin-Ciocalteu reagent (Merck KGaA, Darmstadt, Germany). After 30 min, 80 µl of Na_2_CO_3_ (7%) was subsequently added to the mixture. Subsequently, the sample absorbance was measured utilizing a microplate reader (Tecan/Sunrise Microplate Reader, Switzerland) at 750 nm against a standard graph for gallic acid (Sigma-Aldrich, St Louis, MO, USA). Total phenolic contents were expressed as micrograms of gallic acid equivalents per milligram of dry extract (µg GAE/ mg).

### Total flavonoid contents

The flavonoid contents of the extracts were measured using the aluminum chloride (AlCl_3_) method. Adapted from a previous study [53], an aliquot of 30 µl of extract solution (1 mg/ml) was mixed with 10 µl of 10% (w/v) AlCl_3_ solution in distilled water, 10 µl of 1 M potassium acetate (CH_3_CO_2_K), 30 µl of distilled water and 170 µl of absolute ethanol. The mixture was incubated for 30 min at room temperature followed by the measurement of absorbance at 415 nm (Tecan/Sunrise Microplate Reader, Switzerland). The values were against a standard quercetin (10–500 µg/ml) which was purchased from Sigma-Aldrich (St Louis, MO, USA). The flavonoid contents were expressed as microgram of quercetin equivalents per milligram (μg QE/mg) of dry extract.

### Antioxidant assessments by ferric reducing antioxidant power (FRAP) analysis

A previous study served as the basis for the adapted experiment [53]. Briefly, ascorbic acid was employed to set the standard calibration curve. Fresh FRAP reagent were prepared by combining 10:1:1 ratio of 0.25 M acetate buffer (pH 3.6), 10 mM 2,4,6-Tripyridyltriazine (TPTZ) (Sigma-Aldrich, St Louis, MO, USA), and 20 mM Ferric chloride (Merck KGaA, Darmstadt, Germany). Then, 18 μl of the extract were mixed with 182 μl of FRAP reagent and incubated at 37 °C for 30 min. The absorbance at 593 nm was measured (Tecan/Sunrise Microplate Reader, Switzerland). Antioxidant activity was calculated in micrograms of ascorbic acid equivalent per milligram dry weight (μg AAE/mg).

### Radical scavenging activity by the DPPH (1,1-diphenyl-2-picrylhydrazyl) assay

In a brief, 20 μl of extract was mixed with 180 μl of a freshly prepared 0.1 mM DPPH reagent (Sigma Aldrich, St Louis, MO, USA). This reagent was made by dissolving 0.001 g of DPPH in 25 ml of absolute EtOH. The mixture was left at room temperature for 30 min after being gently shaken for 2 min. The absorbance was measured with a microplate reader (Tecan/Sunrise Microplate Reader, Switzerland) at 517 nm. The percentage of radical scavenging effect was calculated by following equation:

$$\mathrm{Percentage}\;\mathrm{of}\;\mathrm{scavering}\;\mathrm{effect}\;\left(\%\right)=\left({\text{A}}_\text{b}-{\text{A}}_\text{s}\right)/{\text{A}}_\text{b}\times100$$

where A_b_ is the absorbance of reagent blank and A_s_ is the absorbance of the reaction with the extract.

Different sample concentrations were utilized to generate antiradical curves, which were used to calculate the EC_50_ values (the concentration necessary to provide a 50% antioxidant activity). Antiradical curves have been created with concentration on the x-axis and scavenging ability on the y-axis. GraphPad Prism version 8.0 for Windows (GraphPad Software, San Diao, CA) was used to calculate the EC_50_ values.

### Cell lines and cell culture

The liver cancer cell line, HepG2 was kindly provided by Dr. Porntip Pinlaor, Associate professor at Faculty of Associated Medical Sciences, Khon Kaen University, Thailand. The cell line was cultured in Eagle's Minimum Essential Medium (EMEM) containing 10% fetal bovine serum and penicillin/streptomycin (100 U/ml and 100 µg/ml) and incubated in a humidified incubator of 5% CO_2_ at 37 °C. For CCA cell lines, KKU-213B (JCRB1556) was developed by Prof. Banchob Sripa at Cholangiocarcinoma Research Institute, Khon Kaen University, Thailand. In our study, KKU-213B cell lines were purchased from Japanese Collection of Research Bioresources (JCRB). For gastric cancer cell lines, AGS was purchased from the American Type Culture Collection (ATCC). A primary normal fibroblast cell line was purchased from American Type Culture Collection (ATCC). KKU-213B, AGS and normal fibroblast were cultured in Ham’s F-12 containing 10% fetal bovine serum and penicillin/streptomycin (100 U/ml and 100 µg/ml) and incubated in a humidified incubator of 5% CO_2_ at 37 °C.

### Cytotoxicity test

The cytotoxicity test was determined by using MTT colorimetric assay. KKU-213B, HepG2 and AGS cells were seeded in 96 well-plate (2,000 cells / well) and incubated for 24 h. Cells were treated with series concentration of crude extracts for 48, and 72 h and incubated at 37 °C in 5% CO_2_ incubator. After 48-, and 72-h treatment, the cells were washed with PBS and incubated with MTT reagent at 37 °C for 2 h. Subsequently, the MTT reagent was removed and DMSO was added to dissolve formazan crystals. All the experiments were reported as mean ± SD of five independent experiments. The levels of crude extract that reduce 50% cell viability (the half maximum inhibitory concentration; IC_50_) were obtained using curves created by plotting cell viability (%) against crude extract concentration. The data were expressed as a percentage of cell viability compared to the untreated control. The general toxicity of normal fibroblast cell line was also included in this assay.

### Flow cytometric assay for apoptosis evaluation

The apoptotic cell distribution was determined using the Alexa Fluor^®^ 488 annexin V/Dead Cell Apoptosis Kit (Invitrogen™, USA) according to the manufacturer’s protocol. Briefly, KKU-213B and AGS cells were seeded in 6 well-plate (200,000 cells/ well) and incubated for 24 h. Cells were treated with 50, 100, and 200 µg/ml of ethyl acetate extract for 48 h and incubated at 37 °C in 5% CO_2_ incubator. After 48 h of crude extracts treatment, the cells were trypsinized and washed with cold PBS. After PBS removal, and cells were resuspended with 100 µl of 1 × annexin binding buffer, 2.5 µl of Alexa Fluor^®^ 488 annexin V, and 1 µl of 100 µg/ml PI. The stained cells were analyzed by flow cytometry (FACS Canto II, BD Biosciences, UK).

### Intracellular reactive oxygen species measurement

Briefly, KKU-213B and AGS cells were seeded into six-well plates in complete Ham’F-12 nutrient medium for 24 h. Then 50, 100, and 200 µg/ml of ethyl acetate extract was added into the cell culture for 48 h. After that, the cells were washed twice using PBS, then incubate in medium containing 5 µM of CM-H_2_DCFDA indicator (Invitrogen™, USA). After 20 min, the cells were washed with PBS and the cell pellets were collected by trypsinization. Five hundred microliter of PI (Invitrogen™, USA) were added before finally examining the intensity of fluorescence by flow cytometry (FACS Canto II, BD Biosciences, UK). The cellular ROS level was determined in duplicate.

### Western blot analysis

The protein extraction from cell pellets was initiated using a RIPA cell lysis buffer. RIPA buffer is prepared by mixing 1.5 ml of NaCl (1 M), 0.1 ml of Triton X, 0.05 ml of Sodium deoxycholate (DOC) (0.5%), 0.01 ml of SDS (0.1%), and 5 ml of Tris (50 mM, pH 7.4), and then bringing the volume up to 10 ml with water. Subsequently, the concentration of the extracted proteins was determined through the application of a Pierce™ BCA Protein assay kit (Pierce Biotechnology, Rockford, IL, USA). To prepare the protein samples, they were heated to 95 °C for 5 min on a hot plate and then rapidly cooled on ice. These treated protein samples were appropriately loaded onto an SDS-PAGE gel with a specific composition, featuring a 4% stacking gel and an 12% separating gel. After gel electrophoresis, the separated proteins were transferred onto a PVDF membrane (Merck, Billerica, MA, USA). The transferred membrane was subject to a blocking step in a solution containing 5% skim milk in Tris-buffered saline supplemented with 0.1% Tween-20 (TTBS) at room temperature for a duration of 1 h. Subsequently, the blocked membrane was subjected to an overnight incubation with primary antibodies at 4 °C. The primary antibodies employed in this study included a rabbit anti-Bcl-2 polyclonal antibody at a 1:1,000 dilution (Proteintech, USA), and a mouse anti-β actin monoclonal antibody at a 1:20,000 dilution (Sigma-Aldrich, USA). After primary antibody incubation, the membranes were thoroughly rinsed with TTBS and incubated with peroxidase-labeled secondary antibodies for 1 h. Following this, a final round of washing with TTBS was carried out to remove unbound secondary antibodies. Ultimately, the membranes were exposed to an Amersham™ ECL™ Prime Western Blot Detection Reagent (GE Healthcare, Buckinghamshire, UK) for chemiluminescence detection, and the ensuing signals were documented utilizing an ChemiDoc MP, Bio-Rad Laboratories Ltd.

### Sample preparation for ^1^H‑NMR analysis

One-hundred mg of plant extracts were placed into a test tube. One ml of Dimethyl sulfoxide-D6 containing 0.1 vol% TMS (Merck KGaA, Cat no.103587) was added and vortexed for 1 h at room temperature. After shaking, the mixture was passed through a 0.20 µm filter (Corning, USA). The supernatant was immediately transferred into a 5 mm NMR tube. Each extract and quality control were tested in 5 replication tubes before being analyzed with NMR. The sample characterization was performed by acquiring ^1^H-NMR 400 MHz (Avance, Bruker BioSpin GmbH, Rheinstetten, Germany). The zg30 pulse sequence applied with water signal suppression and 32 scans at 27 °C.

### ^1^H-NMR based metabolomics analysis

^1^H-NMR spectra regions between lower–0.5, and 8–upper were excluded before analysis due to the residual peaks of solvents (standard TMS, and downfield noise signals). NMR spectral data were normalized by probabilistic quotient normalization method and aligned using CluPA method in R statistical software using the ASICS package combined with in-house R scripts. The mean centered and UV scaling procedures were applied to the data using the Metabom8 package (v. 0.4.4, https://github.com/tkimhofer/metabom8, accessed on 16/06/2023), for multivariate data analyses, to attenuate the effect of dominant variables and noise while amplifying weak signals to the largest possible. Principal component analysis (PCA) and supervised partial least squares regression analysis (PLS) were performed. The resonances of interest were investigated using statistical total correlation spectroscopy (STOCSY) and compared to online metabolite databases such as biological magnetic resonance data bank (BMRD), The Natural Product Magnetic Resonance Database (NPMRD), human metabolome database (HMDB) and Chenomx software.

### Statistical analysis

IBM SPSS V.23.0 statistical package (SPSS Inc., Chicago, IL) was used for statistical analysis. The quantitative bar chart for functional analysis, the EC_50_ values and the dose–response curve for IC_50_ was calculated and created by GraphPad Prism version 8.0 for windows (GraphPad software, San Diao, CA). The statistical significance between different groups was detected by one-way ANOVA. *P* < 0.05 was considered statistically significant.

## Results

### Quantitative phytochemical analysis and antioxidant activity

The total phenolic and total flavonoid contents of *Shorea roxburghii* extracts were shown in Table 1. The total phenolic content was 213.43 ± 6.94 µg GAE/mg extract in the ethyl acetate extract, which was higher than those of the ethanol, water, and hexane extract. The ethyl acetate extract showed the maximum value for the total flavonoid contents (127.52 ± 14.58 µg QE/mg) followed by ethanol and water extract, while the hexane extract showed the minimum value (9.56 ± 0.02 µg QE/mg). One-way ANOVA indicated that the ethyl acetate, ethanol, water, and hexane extract values were significantly different (*P* < 0.05) for the total phenolic content, and *P* < 0.05 for the total flavonoid content in the extracts.

**Table 1:** Total phenolic, flavonoid content and its antioxidant accessed by FRAP method

Crude *Shorea roxburghii* extracts	Total phenolic content (µg GAE/ mg dry wt)	Total flavonoid content (µg QE/ mg dry wt)	Ferric reducing power assay (µg AAE/ mg dry wt)
Hexane	1.39 ± 0.65^#^	9.56 ± 0.02^#^	18.93 ± 2.33^#^
Ethyl acetate	213.43 ± 6.94^#^	127.52 ± 14.58^#^	68.07 ± 2.49
Ethanol	165.65 ± 2.23^#^	65.63 ± 7.70^#^	66.54 ± 7.05
Water	124.63 ± 4.45^#^	22.60 ± 2.80^#^	68.89 ± 4.93

The data are expressed as the means ± SD

^#^*P* < 0.05 vs. other groups

**Fig. 1:**
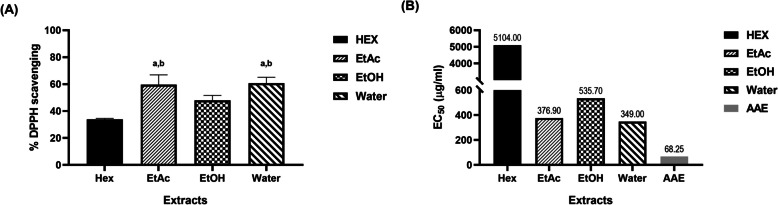
DPPH radical scavenging activity of crude *Shorea roxburghii* extracts. (**A**) DPPH radical scavenging activity of crude *Shorea roxburghii* extracts with 500 μg/ml (^a^*P* < 0.05 compared with ethanol, ^b^*P* < 0.05 compared with hexane) (**B**) EC_50_ values of DPPH scavenging effect in crude *Shorea roxburghii* extracts and the standard compound. (HEX; Hexane, EtAc; Ethyl acetate, EtOH: Ethanol, Water: Distill water, AAE: Ascorbic acid)

To evaluate the antioxidant capacity, ferric ion reducing antioxidant power (FRAP) and DPPH radical scavenging experiments were performed as shown in Table 1 and Fig. 1, respectively. Water extract displayed reducing power, with FRAP values of 68.89 ± 4.93 µg AAE/mg extract which was slightly higher than ethyl acetate and ethanol (68.07 ± 2.49 and 66.54 ± 7.05 µg AAE/mg, respectively) as shown in Table 1. Nevertheless, a one-way ANOVA revealed that the FRAP values of the water, ethyl acetate, and ethanol extracts did not differ significantly (*P* > 0.05).

For DPPH radical scavenging experiments demonstrates that water extract at 500 µg/ml concentration exhibited DPPH radical scavenging activity with percent inhibition values of 60.70 ± 4.45 (EC_50_: 349 µg/ml) following by ethyl acetate 59.67 ± 7.20 (EC_50_: 376.9 µg/ml), ethanol 47.96 ± 3.69 (EC_50_: 535.7 µg/ml) and hexane 33.94 ± 0.72 (EC_50_: 5104 µg/ml) as shown in Fig. 1. There were no significant differences between DPPH values of water and ethyl acetate extracts (*P* > 0.05) according to one-way ANOVA. However, water and ethyl acetate extracts showed significantly higher DPPH inhibition values when compared with ethanol and hexane extracts (*P* < 0.05).

### Cytotoxicity test

**Fig. 2:**
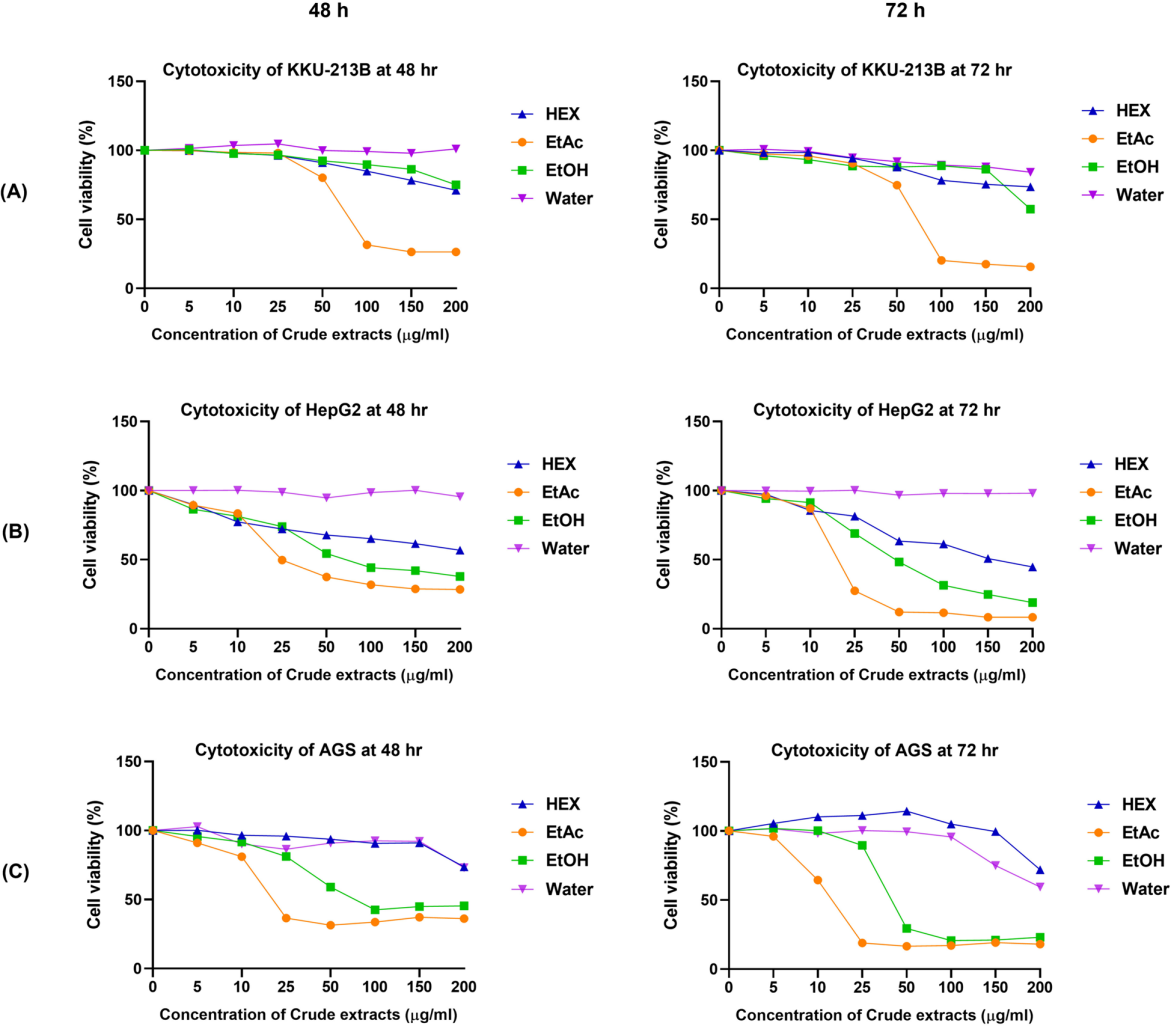
Cytotoxicity of *Shorea roxburghii* extracts on gastrointestinal cancer cell lines. Gastrointestinal cancer cell lines: (**A**) KKU-213B, (**B**) HepG2, (**C**) AGS treated with four crude *Shorea roxburghii* extracts with a series concentration 0–200 µg/ml in 48 and 72 h (HEX; Hexane, EtAc; Ethyl acetate, EtOH: Ethanol, Water: Distill water)

**Fig. 3:**
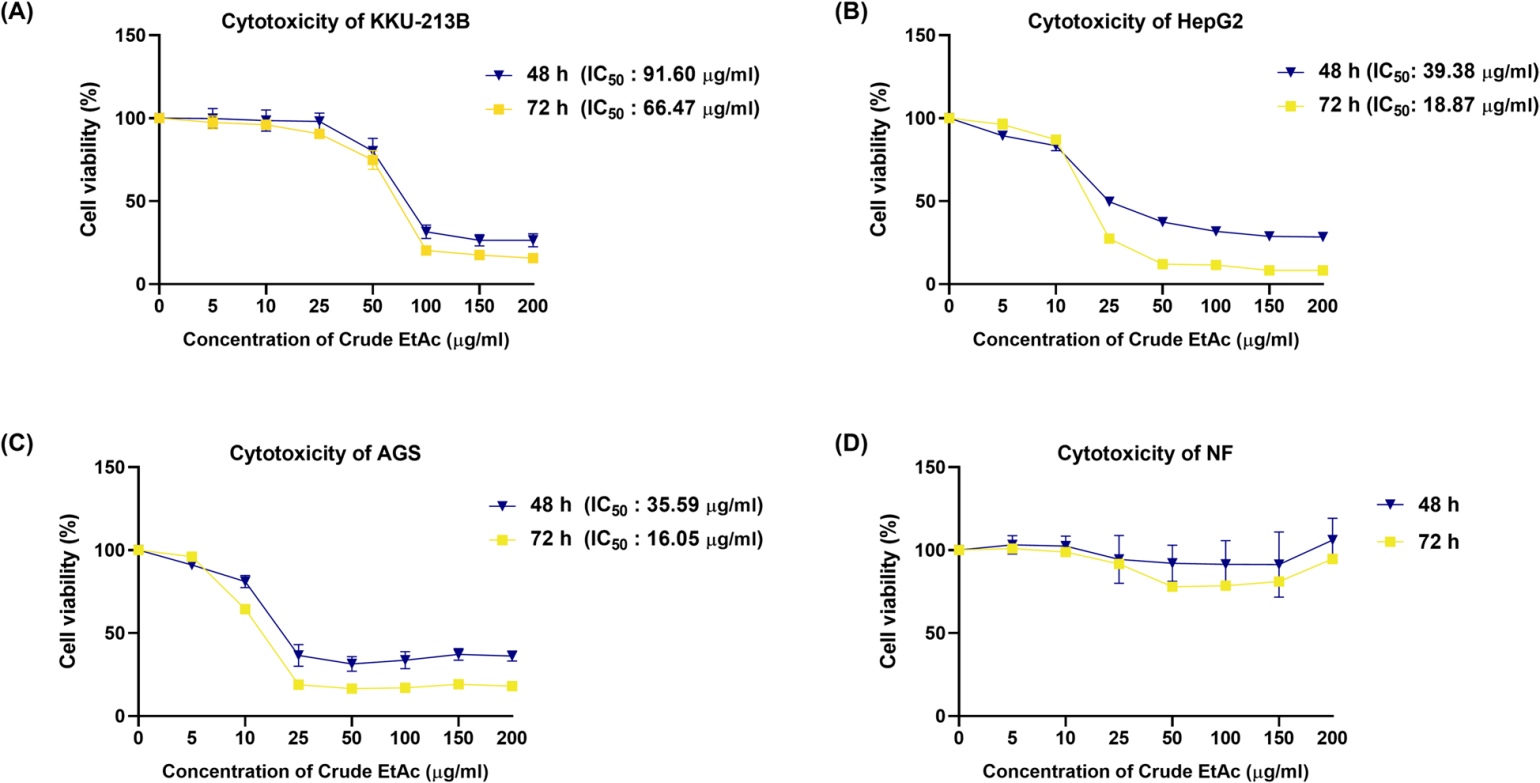
Cytotoxicity of ethyl acetate extract of *Shorea roxburghii* on gastrointestinal cancer cell lines and normal fibroblast. Gastrointestinal cancer cell lines: (**A**) KKU-213B, (**B**) HepG2, (**C**) AGS treated with crude ethyl acetated of *Shorea roxburghii* extracts with a series concentration 0–200 µg/ml in 48 and 72 h compared with (**D**) normal fibroblast cell. (EtAc; Ethyl acetate)

To test for the cytotoxic effects of *Shorea roxburghii* extracts, three types of gastrointestinal cancer cells including KKU-213B, HepG2 and AGS cells were selected. All cell lines were treated with a concentration range of 0–200 µg/ml of crude extracts in vitro for 48 and 72 h, and the cell viability was determined by performing an MTT assay. The results were represented in Fig. 2. Our results demonstrated that crude ethyl acetate extract has the greatest inhibitory effect on gastrointestinal cancer cell growth compared with other extracts. After 48 h of treatment, ethyl acetate extract inhibited the growth of KKU-213B, HepG2, and AGS with half maximal inhibitory concentrations (IC_50_) of 91.60 µg/ml, 39.38 µg/ml, and 35.59 µg/ml, respectively and IC_50_ was determined to be 66.47 µg/ml, 18.87 µg/ml, and 16.05 µg/ml after 72 h of treatment as shown in Fig. 3. Even at the same concentrations, ethyl acetate extract inhibited the growth of normal fibroblast cells only slightly. For further functional analysis, ethyl acetate extract was chosen.

### Crude ethyl acetate of *Shorea roxburghii* extract induce intracellular ROS and apoptosis

**Fig. 4:**
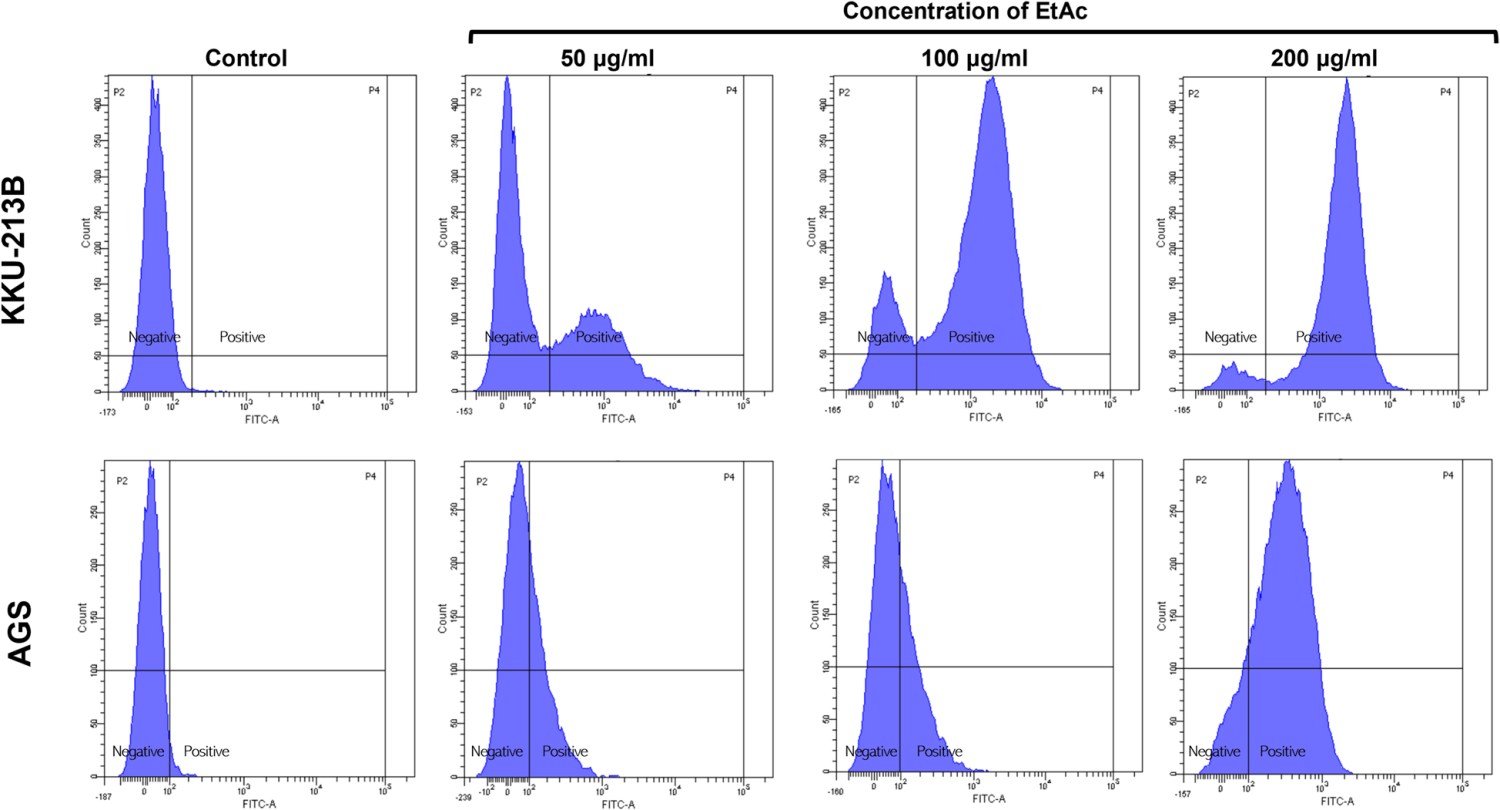
Crude ethyl acetate of *Shorea roxburghii* extract induce intracellular ROS. KKU-213B and AGS cells were treated with crude ethyl acetate of *Shorea roxburghii* extract for 48 h, and intracellular ROS level was determined by flow cytometry. (EtAc; Ethyl acetate)

**Fig. 5:**
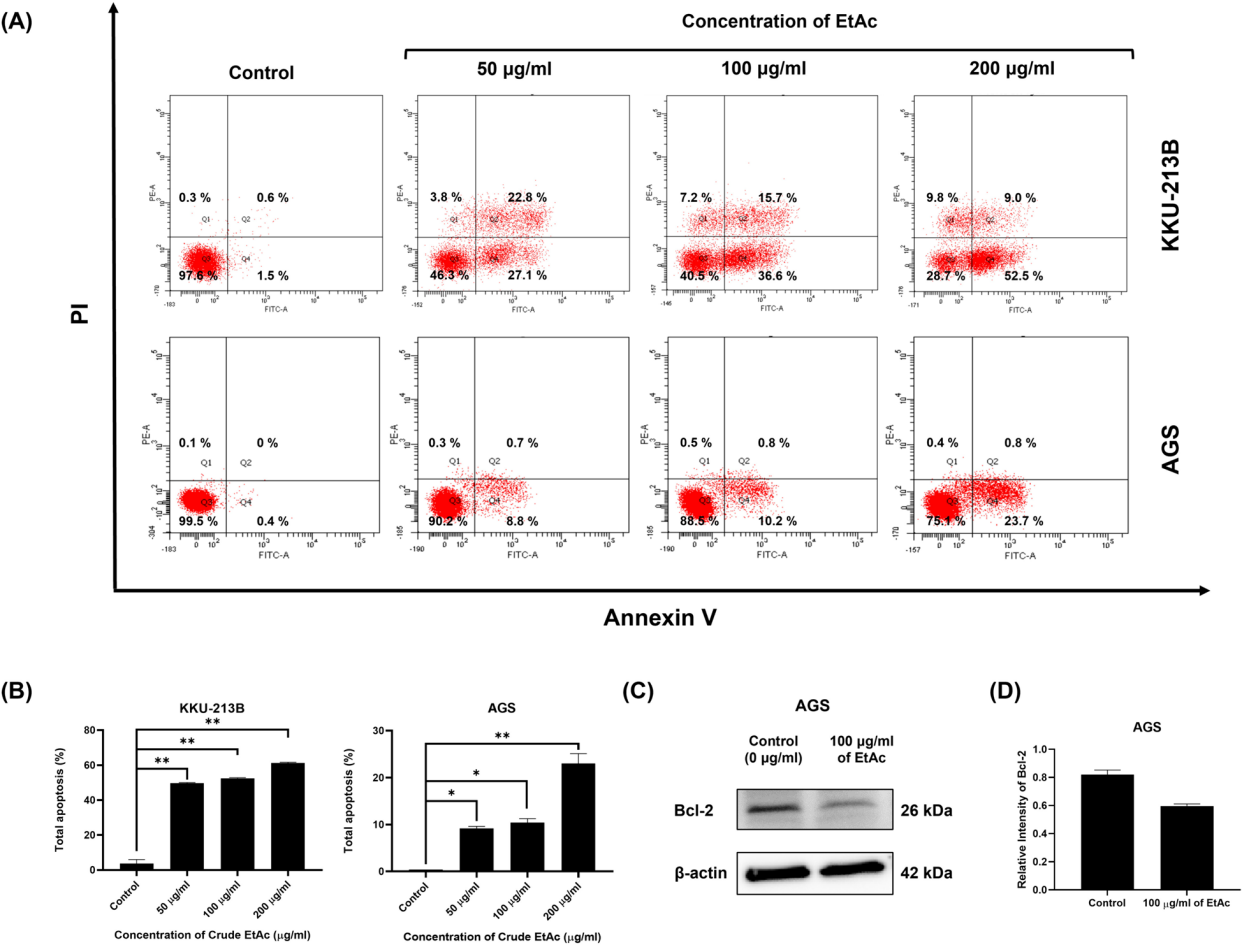
Crude ethyl acetate of *Shorea roxburghii* extract induce apoptosis. (**A**) KKU-213B and AGS cells were treated with crude ethyl acetate of *Shorea roxburghii* extract for 48 h, and apoptosis was determined by flow cytometry using Annexin V/PI double staining. (**B**) Quantitative analysis of apoptosis is shown. (*P < 0.05, **P < 0.001 versus control.) (**C**) Western blot analysis was performed to assess the expression levels of Bcl-2 after treatment in AGS and (**D**) Quantitative analysis of Bcl-2 expression is shown. Original images of blots are shown in Fig. S1. (EtAc; Ethyl acetate)

The two types of gastrointestinal cancer cells, KKU-213B and AGS, were chosen for additional studies into intracellular ROS and apoptosis changes induced by crude ethyl acetate. Figure 4 demonstrates that after 48 h of ethyl acetate extract treatment, the intensity of fluorescence was increased in 100, and 200 µg/ml of treatment in KKU-213B and 200 µg/ml in AGS compared with untreated control. This result reveal that ethyl acetate extract induced intracellular ROS levels in both cancer cells in a dose-dependent manner. In addition, we determined whether the ethyl acetate extract inhibited the growth of gastrointestinal cancer cells by inducing apoptosis. Both cells were treated with crude ethyl acetate and stained with Annexin V-FITC/PI for flow cytometry analysis. After 48 h of treatment at 50, 100, and 200 µg/ml, the rates of apoptosis in KKU-213B were (49.65 ± 0.35) %, (52.50 ± 0.28) %, (61.25 ± 0.35) % and the rates of apoptosis in AGS were (9.20 ± 0.42) %, (10.40 ± 0.85) %, (23.00 ± 2.12) %, respectively. The results indicated that after treatment with crude ethyl acetate of *Shorea roxburghii* extract both cells had significant increases in cellular apoptosis, which are shown in Fig. 5A and B. In contrast, cells treated with 0.2% DMSO exhibited normal cell viability with minimal cell death (3.70 ± 2.26) % for KKU-213B and (0.40) % for AGS. In addition, the expression of apoptosis-related proteins was investigated using western blot analysis. As shown in Fig. 5C and D, the expression of Bcl-2 was decreased in the AGS cell line.

### Identification of anticancer activity-related bioactive compounds

**Fig. 6:**
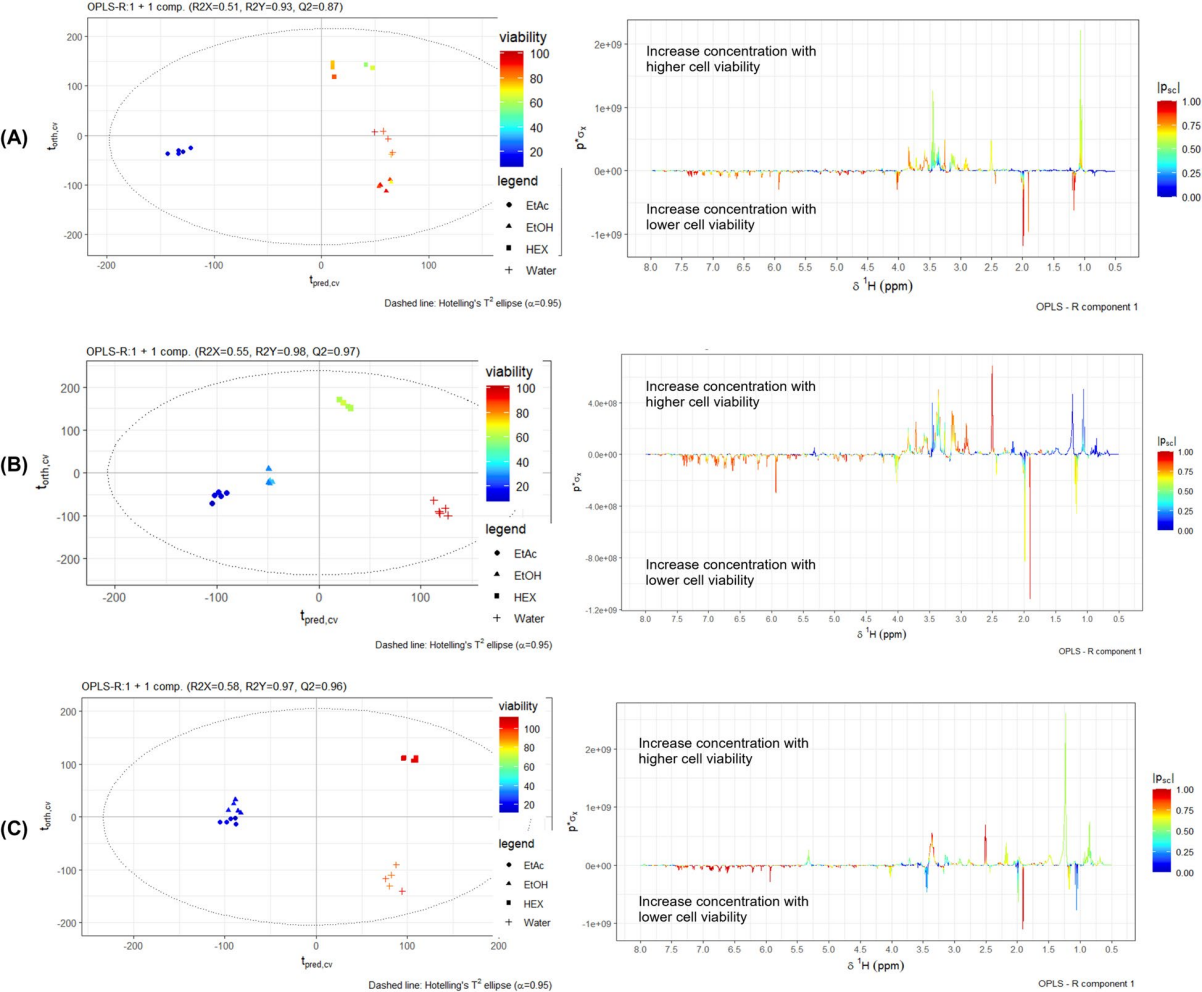
Gastrointestinal cancer cell viability-related bioactive compounds analyze by ^1^H-NMR. OPLS scores and corresponding coefficient loading plots displaying significant metabolites with cell viability on (**A**) KKU-213B, (**B**) HepG2 and (**C**) AGS. (HEX; Hexane, EtAc; Ethyl acetate, EtOH: Ethanol, Water: Distill water, AAE: Ascorbic acid)

All crude extracts of *Shorea roxburghii* flower were performed by ^1^H-NMR to investigate the gastrointestinal cancer cells viability reduction-related bioactive compounds. The ^1^H-NMR data sets of crude *Shorea roxburghii* extracts and percentage of cell viability were analyzed using partial least square (PLS) regression analysis. The validity of the regression models was determined using the goodness of fit (R^2^) and the goodness of prediction (Q^2^) of above 0.6 [54]. Interestingly, plots of the PLS scores indicated that the decreasing of gastrointestinal cancer cells was related to the crude ethyl acetate extract of *Shorea roxburghii* as shown in Fig. 6. According to three cancer models, the liver cancer model was the highest goodness of prediction with Q^2^ = 0.97. The common key metabolites found in three types of gastrointestinal cancer models included acetic acid, resveratrol, ferulic acid, fumarate, catechin, gallic acid, m-coumaric acid, cinnamic acid, folate as shown in Table 2.

**Table 2:** Common compounds in crude *Shorea roxburghii* extracts correlated with GIC cancer cell line inhibition

Metabolites	Chemical shift
Acetic acid	1.909 (s)
Resveratrol	6.196 (m); 6.405 (d); 6.715 (m); 6.903(s); 7.141 (d)
Ferulic acid	6.405 (d); 6.903 (d); 7.306 (d)
Fumarate	6.529 (s)
Catechin	4.569 (d); 6.715 (m); 7.017 (d)
Gallic acid	7.039 (s)
m-Coumaric acid	6.444 (d); 7.141 (d); 7.345 (m)
Cinnamic acid	7.345 (m); 7.776 (s); 7.798 (s)
Folate	7.744 (d); 8.031 (d)

*s* singlet, *d* doublet, *t* triplet, *m* multiplet

## Discussion

Natural products contain substances with varied pharmacological characteristics that have the potential to be used in the research and development of therapeutics. Over 50,000 phytochemical substances including alkaloids, bibenzyls, phenanthrenes, stilbenoids, phenols, flavonoids, anthocyanins, and polysaccharides have been found in plants [55]. These substances have antioxidant, anti-inflammatory, and antibacterial properties, as well as the ability to prevent cancer progression and induced cancer cell death.

*Shorea roxburghii* is a medicinal plant that found in all regions of Thailand. Some parts of it have been reported that contain various a large number of effective compounds. *Shorea roxburghii* leaves extract has demonstrated its ability to protect against CTX-mediated kidney damage due to its antioxidant and anti-inflammatory properties [56]. Oligostilbenoids isolated from the bark of *Shorea roxburghii* show the anti-cancer effect in melanoma [48]. Moreover, two compounds which isolated from the roots of *Shorea roxburghii* demonstrated strong toxicity against KB and HeLa cell lines with IC_50_ values of 6.5, 8.5 and 8.7, 10.1 μg/ml, respectively [57]. Nevertheless, no documented studies have been conducted to investigate the biological abilities or pharmacological effects of the *Shorea roxburghii* flower. We have chosen to focus on the flower of *Shorea roxburghii* extract due to its underexplored active compounds. Flowers are easier to harvest, and process compared to other plant parts. They possess significant economic value and are utilized in various industries, including beneficial tea, and traditional medicine. This offers an opportunity to discover novel anticancer properties and enhance feasibility for drug development and clinical trials. In this study, the flower of *Shorea roxburghii*, was collected and extracted in order to examine its phytochemical, antioxidant, and anticancer properties on gastrointestinal cancer (GIC).

Normally, aerobic metabolism which is mediated by mitochondrial electron transfer leads to the generation of the endogenous reactive oxygen species (ROS) [58]. Intracellular ROS reduction is controlled by endogenous antioxidant enzymes such as glutathione peroxidases, catalases, and superoxide dismutases [59]. However, antioxidant defense systems may not be sufficient to maintain redox equilibrium which is one key to cancer progression. Exogenous antioxidants are important in the role of cancer prevention and treatment. Our study investigated two groups of phytochemical contents that have antioxidant ability: phenolic and flavonoid. The result revealed that ethyl acetate extract of *Shorea roxburghii* showed the maximum value for the total phenolic and flavonoid contents. Two antioxidant assessment methods, namely the DPPH test and the FRAP assay, were used to evaluate the ability of crude *Shorea roxburghii* extracts in terms of electron donation to the stable DPPH free radical and the reduction of ferric (Fe^3+^) to ferrous (Fe^2+^) ions [60]. Our result revealed that water and ethyl acetate extracts were the highest antioxidant activity with the lowest EC_50_ values in DPPH. Ethanol extract possessed antioxidant properties, as evidenced by FRAP values that demonstrated no significant difference between water and ethyl acetate extract. It revealed that antioxidants properties were found in three crude extracts except in hexane extracts. Investigated by DPPH assay, three crude extracts of *Shorea roxburghii* (EC_50_: 349—535.7 µg/ml) have higher antioxidants activities compared with EC_50_ in other Thai medicinal plants such as *Clausena excavata* (904.53 ± 3.23 µg/ml), *C. harmandiana* (2037.66 ± 39.23 µg/ml), *Trichosanthes anguina* (869.95 ± 1.53 µg/ml) [61, 62]. Our findings highlight *Shorea roxburghii* as a rich source of compounds with potent antioxidant activity compared to others. Conversely, water extract seems to have the highest antioxidant activity but less phenolic and flavonoid contents when compared with ethyl acetate and ethanol extract. The potential antioxidant ability of the water extract might be related to other kinds of phytochemicals that were not assessed in this study.

On the basis of dose, the role of antioxidants in cancer can be divided into two categories: cancer prevention and cancer treatment. Antioxidants should be low for cancer prevention, but they can protect normal cells and do not transform them into cancer cells. On the other hand, antioxidants should be high in therapeutic doses to inhibit cancer cell growth while remaining non-toxic to normal cells. From our result, we suggest that water extract should be used in cancer prevention due to it having the highest antioxidant activity with the lowest dose. Moreover, it can be used as a consumed manufactured drink which is easy to prepare compared to other extracts. There were many manufactured drinks from plants that have been used in cancer prevention, for instance, the extract of *Camellia sinesis* (green tea) and cocoa from *Theobroma cacao* (chocolate) [63–65].

In the role of cancer treatment, gastrointestinal cancer patients are typically discovered at an advanced stage or have a high recurrence rate after surgery, resulting in a few effective therapy options. Chemotherapy is recommended for gastrointestinal patients. Toxicity and severe adverse effects are significant disadvantages of chemotherapeutic cancer treatment strategies [66–70]. To overcome these limitations, the discovery of new anticancer agents which have fewer side effects is a challenge in cancer treatment. In this study, the cytotoxicity effect of four crude extracts of *Shorea roxburghii* were evaluated on three different gastrointestincal cancer cell lines: KKU-213B, HepG2 and AGS by MTT assay. Each crude extract exhibited a different anti-cancer activity as they inhibited the viability of the gastrointestinal cancer cell lines. The IC_50_ values varied among the extracts revealing the influence of the bioactive compound constituents composed within them. The ethyl acetate extract exhibited the highest level of inhibition in three distinct types of gastrointestinal cancer cell lines, while demonstrating slightly less toxicity in normal fibroblast cells. It inhibited AGS cell proliferation with IC_50_ of 35.59 µg/ml, which was only half the concentration required compared to the ethyl acetate extract of *Celastrus orbiculatus* (IC_50_ 68.24 mg/l or equivalent to 68.24 µg/ml) [71]. Previous research reported that the ethanolic extract of Thai noni juice Chiangrai (*Morinda citrifolia*) reduced viability KKU-213B cells with IC_50_ values of 2.14 ± 0.08 mg/ml for 48 h, which was higher than 20-fold compared with ethyl acetate extract of *Shorea roxburghii* (91.60 µg/ml or equivalent to 0.09 mg/ml) [72]. After 48 h of treatment, ethyl acetate extract inhibited cell viability of HepG2 cells with IC_50_ of 39.38 µg/ml, whereas other medicinal plant *Thymus daenensis* and *Stachys pilifera* showed higher IC_50_ values of 210.2 ± 12 µg/ml and 109.7 ± 5 µg/ml, respectively [73].

Cancer cells proliferate uncontrollably, resulting in higher ROS levels than normal cells, thus indicating that cancer cells have more antioxidative ability and ROS tolerance than normal cells. Therefore, increasing ROS over the cytotoxic threshold can disrupt redox equilibrium and result in cancer cell death [74]. Our study found that ethyl acetate extract treatment-induced ROS generation in KKU-213B and AGS cells in dose dependent. In addition, Annexin V-FITC/PI double staining showed a concentration-dependent increase in the apoptotic rate of KKU-213B and AGS cells. The results suggested that the secondary metabolites present in crude ethyl acetate of *Shorea roxburghii* induce ROS and therefore resulting in apoptosis of KKU-213B and AGS cells. Moreover, the expression of Bcl-2 was examined in AGS cells treated with crude ethyl acetate to investigate apoptotic protein expression. The decrease in Bcl-2 confirmed that crude ethyl acetate triggered apoptotic cell death in AGS. Support our finding in the role of anti-cancer, oligostilbenoids isolated from the bark of *Shorea roxburghii* also possessed antiproliferative action against SK-MEL-28 melanoma cells through the arrested cell division cycle at the G1 phase [48].

The profiling of candidate bioactive compounds in four crudes of *Shorea roxburghii* were then evaluated using ^1^H-NMR metabolomic data analysis. The PLS score plot revealed that ethyl acetate extract was strongly correlated with decreasing gastrointestinal cell viability. Resveratrol, a compound that possesses various biological activity including anticancer activity, was the one common compound that we found in three cancer models. Previous research has provided support of results in our study that resveratrol is also found in the acetone extract of the roots of *Shorea roxburghii* [57, 75]. Anticancer activity of resveratrol has been reported in vitro*, *in vivo and in clinical trials. Resveratrol induced apoptosis in gastric cancer cells via NF-κB down-regulation leading to a decrease in the level of anti-apoptotic factor Bcl-2 and an increase in apoptotic factors caspase-3 and caspase-8 [76]. Resveratrol inhibited doxorubicin resistance in gastric cancer cells by stimulation of PTEN/Akt signaling pathway [77]. In addition, the results of Howells et al. demonstrated a significantly increased in the expression of cleaved caspase-3, apoptotic marker, in colorectal cancer patient’s tissue after treatment with pure resveratrol compared with placebo [41]. Interestingly, we discovered a correlation between the group of cinnamic acid and its derivative, ferulic acid, and the decrease in cell viability in all tested cell lines. To support our findings, it has been demonstrated that ferulic acid can induce cytotoxicity in many tumor cell lines, including human osteosarcoma, prostate, and cervical cancers [78–80]. It has been reported that ferulic acid induces cytotoxic effects on HepG2 cells by activating caspases-8 and caspase-9 [81]. Das et al. demonstrated that ferulic acid can initially eliminate intracellular ROS in HepG2 cancer cells[82]. Furthermore, ferulic acid suppresses cell viability, impedes cell cycle progression, and increases the rate of apoptosis in human colon adenocarcinoma cells (HT-29) [83]. In addition, the doublets (d), doublets (d) and multiplets (m) at 6.444, 7.141, and 7.345 ppm, respectively, were the protons in m-coumaric acid [84]. Coumaric acid is a phenolic compound that is a hydroxy derivative of cinnamic acid [85]. There are three isoforms of coumaric acid consist of o-coumaric acid, m-coumaric acid, and p-coumaric acid [86, 87]. A previous study reported that m- and p- coumaric acid has a role in anti-oxidant ability [88]. Several investigations have demonstrated that p-coumaric acid possesses anti-cancer capabilities whereas the potential anti-cancer effects of m-coumaric acid remain uncertain [89]. Our study provides for the first time evidence of potential compounds with anti-cancer properties derived from the flower of *Shorea roxburghii*. The underlying mechanisms by which the candidate bioactive compounds in the flower of *Shorea roxburghii* inhibit the growth of cancer cells require additional investigation.

## Conclusion

The findings of this study provide evidence supporting the possibility of crude derived from *Shorea roxburghii* as a potential source of bioactive compounds with antioxidant and anticancer properties. This provides a basis for future comprehensive studies of natural resources in a sustainable manner and discover more potent and safer anticancer agents. Additional well-designed animal model experiments and clinical trials are required to confirm the efficacy of its action.

### Supplementary Information

**Supplementary Material 1.**

## Data Availability

The data that support the findings of this study are available from the corresponding author upon reasonable request.

## Abbreviations

DPPH: 1,1-Diphenyl-2-picrylhydrazyl
MTT: 3-(4,5-Dimethylthiazolyl-2-yl)-2,5-diphenyl tetrazolium bromide
GAE: Gallic acid equivalents
QE: Quercetin equivalents
AAE: Ascorbic acid equivalent
HEX: Hexane
EtAc: Ethyl acetate
EtOH: Ethanol
Water: Distilled water
ROS: Reactive oxygen species
DMSO: Dimethyl sulfoxide
ANOVA: One way analysis of variance
