# Supplementary material for: Anticancer properties and metabolomic profiling of Shorea roxburghii extracts toward gastrointestinal cancer cell lines
Source: BMC Complement Med Ther. 2024 Apr 30;24:178. doi: 10.1186/s12906-024-04479-1 (PMC11061966; doi:10.1186/s12906-024-04479-1)
Supplement: Supplementary file 1 — Supplementary Material 1. [file 12906_2024_4479_MOESM1_ESM.pdf]

Figure S1: Original images of blots shown in Figure 5C

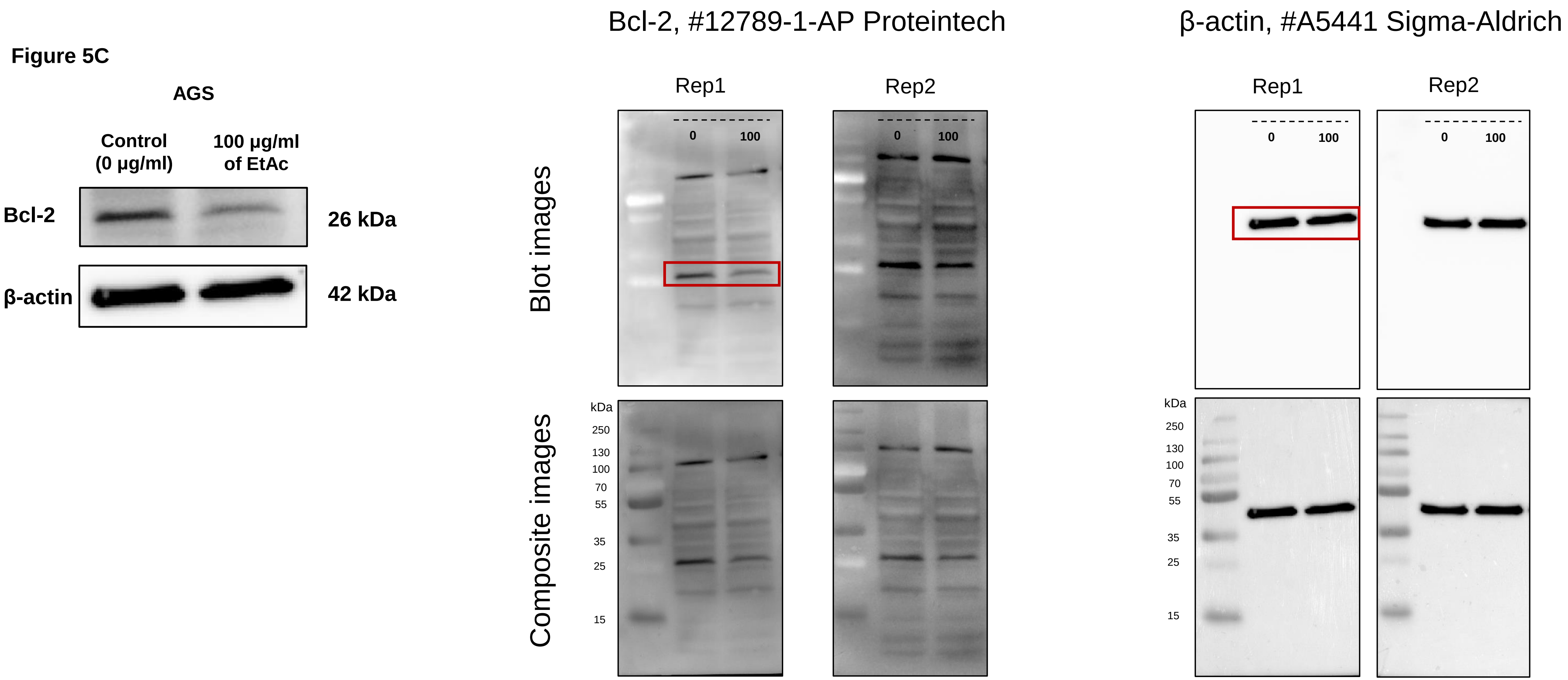

Boxed bands are shown in Figure 5C as representative.
